# Improved strategy for jet‐in‐air cell sorting with high purity, yield, viability, and genome stability

**DOI:** 10.1002/2211-5463.13248

**Published:** 2021-07-30

**Authors:** Xinghui Song, Jiajia Wang, Yanwei Li, Yueting Xing, Chun Guo, Yingying Huang, Lintao Xu, Hu Hu, Lin‐lin Wang

**Affiliations:** ^1^ Core Facilities Zhejiang University School of Medicine Hangzhou China; ^2^ Department of Neurosurgery Second Affiliated Hosptial of Zhejiang University School of Medicine Hangzhou China; ^3^ Department of Basic Medicine Sciences Zhejiang University School of Medicine Hangzhou China; ^4^ Department of Basic Medicine Sciences, and Department of Orthopaedics of Sir Run Run Shaw Hospital Zhejiang University School of Medicine Hangzhou China

**Keywords:** drop delay, gene expression, jet‐in‐air cell sorting, single‐cell sequencing, viability, yield

## Abstract

Flow cytometric sorting is a vital tool in biological research and clinical diagnostics. Theoretically, a high‐speed jet‐in‐air sorter is a fluorescent‐activated cell sorting sorter that ideally processes cells with high purity, yield, and viability. However, high‐speed jet‐in‐air sorting is a complex process due to its inherent requirements for high fluidic stability and electronic and timing precision. Here, we report that an additional manual correction of drop delay leads to improved cell yield. Adding 2% FBS to the loading buffer had no significant effect on the fate of sorted cells in 4 h. However, the addition of a suitable concentration of FBS/BSA in the collecting buffer resulted in a notable increase in cell count and proliferation and a significant decrease in cell apoptosis for cell lines and primary cells. Moreover, the level of gene expression remained steady in the 5% FBS collecting buffer. In summary, here we demonstrate techniques that can be easily followed to refine sorted yields of healthy cells.

## Graphical abstract

Flow cytometric sorting is a vital method in biological research and clinical diagnostics. Obtaining high‐quality sorted cells is an ongoing problem. Here, we demonstrate that a certain concentration of FBS helps to increase cell yield and cell viability and keep gene expression stable. Our work provides an effective sorting strategy.

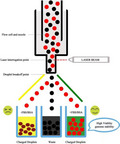

**Abbreviations**
CFSE: carboxyfluorescein diacetate
CSC: central sheath catch
DMEM: Dulbecco's modified Eagle's medium
FACS: fluorescent‐activated cell sorting

Flow cytometric sorting is a vital tool in biological research and clinical diagnostics [1]. This method functions not only as a technology for isolating target cells but can also provide abundant information on targeted cells, which can help to guide subsequent experiments [2, 3, 4]. Cell sorting is indispensable because heterogeneous cell suspensions can be purified into fractions containing single cell types based upon virtually unlimited combinations of user‐defined parameters [5]. Therefore, the cell sorter is the foundation for numerous downstream studies.

Theoretically, a high‐speed jet‐in‐air sorter is a fluorescent‐activated cell sorting (FACS) sorter that ideally processes cells with high purity, yield, and viability [6]. However, high‐speed jet‐in‐air sorting is a complex process due to its inherent requirements for high fluidic stability and electronic and timing precision. Therefore, a highly accurate sorting strategy to achieve high yield and good biocompatibility, including maintaining high viability and functionality, is a considerable challenge facing high‐speed jet‐in‐air sorters and users.

It was determined that effective sorting is highly dependent on appropriate drop delay determination. Incorrect determination of the instrument drop delay affects yield and purity [7]. Meanwhile, the effect of the collecting buffer was examined. Marie *et al*. [8] added BSA to the collecting buffer and notably improved the success of the culture of phytoplankton strains. Maxwell *et al*. [9] demonstrated that the viability and membrane integrity of spermatozoa could be improved by adding seminal plasma into the collecting buffer. Therefore, these results suggest that the more similar the collecting buffer is to the living environment of the sorted cells, the higher the cell viability is. However, there was no clear research about adding BSA or FBS to the collecting buffer during sorting would affect the cell count, apoptosis, proliferation, and gene expression and which concentration of BSA or FBS in the collecting buffer is suitable have not been determined. Moreover, whether adding BSA or FBS in loading buffer would have protective effect during sorting also has not been elucidated.

In this study, we adjusted the drop delay to increase purity and yield. Meanwhile, we investigated the effects on cell count, apoptosis, proliferation, and endogenous gene expression caused by adding different concentrations of FBS or BSA to the loading buffer and the collecting buffer during FACS. Based on the data, we suggested the suitable concentration of FBS or BSA for loading buffer and collecting buffer. This strategy is expected to pave the way for further advances in FACS for signal cells and to facilitate downstream experiments after sorting. The strategy is useful for novice users that want to refine their ability to increase yields of healthy cells.

## Materials and methods

### Beads

Calibration beads for MoFlo Astrios (Beckman Coulter, Inc., Miami, FL, USA) were diluted according to the manufacturer's instructions during the cell sorter setup procedures to maximize the instrument's optical alignment. Flow‐Check™ beads (Beckman Coulter, Inc.) were used directly without dilution during the identification of the instrument drop delays. CS&T research beads (BD Biosciences, San Jose, CA, USA) were used directly without dilution during the identification of the instrument drop delay by the R‐max method.

### Cell culture

Jurkat cells were cultured in RPMI‐1640 medium, and Human ESC‐MSCs and 293T cells were cultured in Dulbecco's modified Eagle's medium (DMEM), supplemented with 10% FBS and 1% penicillin and streptomycin in a 5% (v/v) CO_2_ incubator at 37 °C.

### Isolation of CD45^+^ cells from spleen

The experimental procedures were approved by the Animal Ethics Committee of Zhejiang University and were carried out in accordance with National Institutes of Health guidelines. The animals were housed in a room with temperature (23 °C) and light (12‐h light/dark cycle) control and had free access to water and diet. To isolate CD45^+^ cells, four 8‐week male C57BL/6 mice were euthanized by cervical dislocation. All spleens were dissected and collected into digestive solution containing 0.5 mg·mL^−1^ papain (Worthington, Shatin, Hong Kong, Cat: LS003119) and 0.5 mg·mL^−1^ collagenase type 2 (Worthington, Cat: LS004176) in high‐glucose DMEM for 10 min. Cells were passed through a 40 μm cell strainer (FALCON, Waltham, MA, USA, ref352340) to create single‐cell suspension. Cells were spun down (500 ***g***) at 4 °C for 5 min, and the supernatant was discarded. Cells were suspended in 3 mL red blood cell lysis buffer (BioLegend, San Diego, CA, USA, Cat: 420301) for 3 min, and then, 6 mL PBS was added to stop lysing. Next, cells were spun down (500 ***g***) at 4 °C for 5 min. After the supernatant was discarded, cells were suspended in 200 μL PBS for CD45^+^ staining. To stain the cells for sorting, the cells were incubated with anti‐mouse CD45^+^ monoclonal antibody conjugated with PE/Cyanine7 fluorophores (BioLegend, Cat: 103114) at 4 °C for 45 min. After wash, cells were resuspended in PBS to 10^7^ cells·mL^−1^ for next step experiment.

### CFSE staining and Hoechst 33342 staining

Prior to sorting, cells were suspended in PBS at a concentration of 1 × 10^6^ cells·mL^−1^. Cells were labeled with carboxyfluorescein diacetate (CFSE, C34554, Invitrogen, final concentration 5 μm) at a concentration of 2 × 10^6^ cells·mL^−1^ in RPMI‐1640 or DMEM with 1 μL CFSE for 10 min at room temperature, and the reaction was subsequently stopped by washing three times using RPMI‐1640 or DMEM containing 10% FBS; cells were later suspended in 1 mL PBS, and 1 μL Hoechst 33342 (Sigma, Shanghai, China) was added to the suspension. A mixture of stained cells and unstained cells with a volume ratio of 1 : 1 was diluted in PBS buffer (cell concentration: 10^5^–10^6^ cells·mL^−1^) before sorting.

### Flow cytometry for cell sorting

A MoFlo Astrios EQ flow cytometer (Beckman Coulter, Inc.) is a high‐end jet‐in‐air sorter equipped with a 488 nm laser set to 200 mW at the laser intersection point, and a standard filter setup was used for cell sorting. In this study, the instrument was configured with a 100 μm nozzle at 30 psi (210 kPa) and 49 kHz droplet generation. The sample offset was set at 0.3 psi and was kept constant for the duration of the experiment. Sorting was performed using a ‘purify mode’ with 1 drop envelope deflection sort mode to collect mass cells, and a ‘single cell mode’ with 0.5 drop envelope deflection sort mode to sort single cell into 96‐ or 1536‐well plates, to give the minimal condition of coincidence events [7]. During the sorting process, the sample and collection tubes were kept at 5 °C. Beckman Coulter summit software was used to control the instrument.

### Flow cytometry for cell count and apoptosis analysis

The flow cytometry instruments included CytoFLEX LX (Beckman Coulter, Inc.), LSRFortessa (BD Biosciences), and Novocyte (ACEA Novocyte3000, Palo Alto, CA, USA) equipped with a sampler, which were used to count cells and to examine cells for apoptosis after sorting.

### Setting of the drop delay

There are two methods used to set the drop delay for the MoFlo Astrios EQ sorter. Based on the stability of the fluidics, the automatic drop delay determination was established using an Intellisort with automatic drop delay determination. The manual method modified the automatic drop delay in the manner described in the manufacturer's instructions and articles [8, 9, 10, 11, 12]. According to the operation manual, the estimated drop delay is displayed in Fig. S1. The drop delay diagram in Fig. S1 shows how the puddles (the circles) are deposited on the slide. When all puddles have been created, we remove the slide and inspect the puddles under a fluorescent microscope, determine the puddle (5#) that contains the most beads, and count the beads in the puddles (4# and 6#) adjacent to the puddle that contains the most beads. The criterion of automatic drop delay is that the difference between the number of beads in the puddles adjacent to the target puddle is less than 3%. For example, 4# puddle includes 1 bead and 6# puddle includes 2 beads. The criterion of manual drop delay is that the difference is zero, and in other words, 4# and 6# puddles include no bead.

### Yield assay by fluorescence for beads

**Fig. 1:**
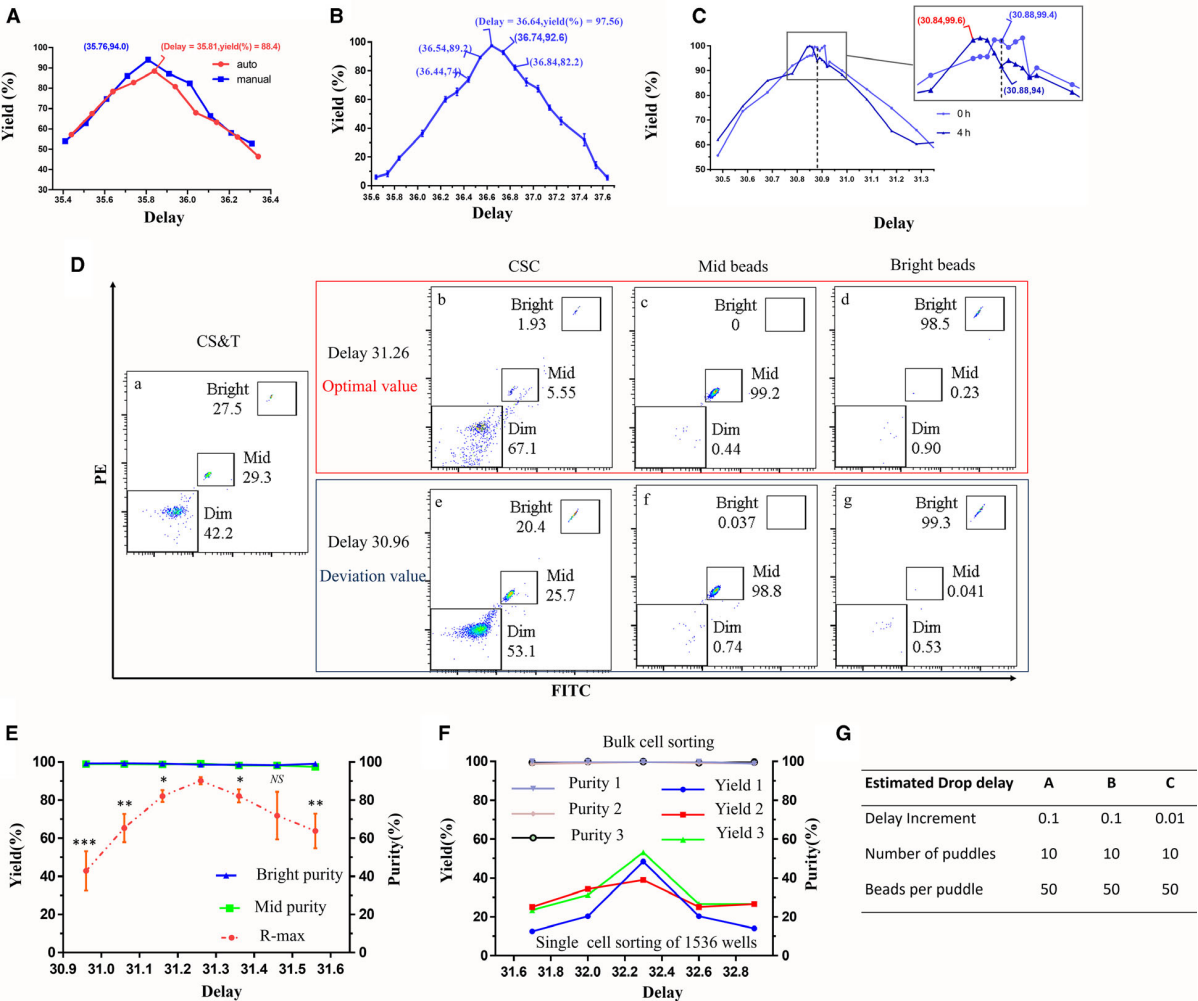
Effects of drop delay on the yield and purity of sorted cells and beads. The effect of drop delay on the yield of beads was tested. Each of the 10 puddles was collected using a drop delay setting with 0.1 intervals. Precisely 50 droplets were deposited into each puddle at different drop delay settings. (A) The effect of drop delay on the yield was tested with automatic and manually adjusted drop delays. The red line is the automatic one, and the blue line is the other one. (B) Map of the correlation between drop delay and yield. (C) Effect of long‐term sorting‐related drop delay deviation on yield. (D, E) The R‐max method was used to assess the effect of drop delay on yield and purity. CS&T beads consist of 3 μm bright or mid and 2 μm dim beads. According to the R‐max method reported, two beads of 3 μm bright or mid and central sheath catch (CSC) were sorted at the same time. After flow cytometry detection, the R‐max value and purity of two kinds of beads were analyzed. (D) Dot plots of flow cytometry detection. (a) Dot plots of CS&T beads. (b–g) Dot plots of CSC, mid and bright beads were sorted with delay value 31.26 (b–d, red frame) and 30.96 (e–g, blue frame). 31.26 is the optimal value. 30.96 is the value of deviation. (F) The yield and purity of cells were assessed under manually adjusted conditions. (G) The estimated drop delay for different experiments of testing drop delay changes. Jurkat cells stained with CFSE and Hoechst 33342 were mixed at 1 : 1 with unstained cells to obtain a suspension (1 × 10^7^ cells·mL^−1^). The double‐positive cells were sorted into 1536‐well plates. Then, each well of cells in the 1536‐well plate was scanned with a BioTek Cytation 1 Cell Imaging Multi‐Mode Reader and imaged individually. The data were analyzed by gen5 3.04 software (BioTek, Winooski, VT, USA). Data are presented as the mean ± SEM. *n* = 3. Each experiment was repeated at least three times. *P < 0.05, **P < 0.01, ***P < 0.001, NS: no significance, by one‐way ANOVA.

The yield according to the setting of drop delay was further investigated. Using automatic drop delay and manually adjusted drop delay separately, 10 different drop delay settings in series were tested with 0.1 intervals. The trend of yield around the optimal drop delay (±0.05) was obtained with 0.01 intervals. All experiments were performed under drop delay Wizard mode, and each drop delay setting was used to sort 50 droplets on glass slides. The estimated drop delay is displayed in the table (Fig. 1G). The number of fluorescent beads in each puddle on each glass slide was counted using a microscope.

### Yield and purity assay by R‐max method for CST beads

According to the R‐max method reported [7], two beads of 3 μm bright and 3 μm mid and central sheath liquid flow were sorted at the same time. After the three kinds of solutions were detected by flow cytometry, the R‐max value and the purity of the two kinds of beads were calculated.

### Yield and purity assay for single cells

For the living cell experiment, we assayed the yield by sorting one cell into each well of 1536‐well plates. Jurkat cells were stained by CFSE and Hoechst 33342. A mixture of stained cells and unstained cells with a volume ratio of 1 : 1 was diluted in PBS buffer (cell concentration: 10^5^–10^6^ cells·mL^−1^). White 1536‐well plates (Corning, Corning, NY, USA) were prepared by adding 5 μL of PBS to each well. After sorting, the plate was removed and centrifuged to keep the cell at the bottom of the well. Each well of a 1536‐well plate was scanned with a BioTek Cytation 1 Cell Imaging Multi‐Mode Reader (BioTek) individually.

The purity assay was performed as follows. CFSE‐stained Jurkat cells were mixed at 1 : 1 with unstained cells to obtain a cell suspension (1 × 10^7^ cell·mL^−1^). Changing the drop delay value, 30 000 negative and positive cells were collected using each different drop delay value. Then, the cell purity was detected by flow cytometry.

### Sorting buffer

Most experiments used 2% FBS in PBS as the loading buffer. Two kinds of loading buffers were tested in this study: PBS and 2% FBS in PBS.

FBS is generally added to the collecting buffer. Because the content of FBS is complex, some experiments are not applicable. In this case, those experiments use BSA instead of FBS. There were three groups in this work: (a) FBS in cell culture medium, with FBS concentrations of 0%, 10%, 50%, and 100% being employed. We used Jurkat, 293T cells, and CD45^+^ cells from mouse spleen; therefore, the culture medium included RPMI‐1640 and DMEM. (b) FBS in PBS, with FBS concentrations of 0%, 5%, and 10% being employed. (c) BSA in PBS, with BSA concentrations of 0%, 0.04%, 0.4%, and 2% being employed.

### Cell count assay

We investigated the effect of the collecting buffer on the actual cell amounts. We tested three kinds of cells: Jurkat cells, 293T cells, and CD45^+^ cells from mouse spleen. We sorted 500 000 cells into different groups of collecting buffer. Fifty microliters of collecting buffer containing sorted cells was directly detected using flow cytometry to count the actual cell amounts. The theoretical cell amounts in 50 μL collecting buffer were calculated based on the cell amounts of 500 000 and the weight difference between the collection tube after sorting and the empty tube. The density of each collecting buffer was considered to be 1 g·mL^−1^. The yield rate is defined as ‘the actual cell amounts/the theoretical cell amounts’.

### Apoptosis assay

The cells were collected by the corresponding collecting buffer. Cell apoptosis was detected immediately after sorting, and the method was described as follows. The supernatant was removed after centrifugation, and Annexin‐V‐FITC/PI staining was performed as previously described [11]. Two‐dimensional flow cytometry was performed to detect early and late apoptotic cells. The percentage of cell apoptosis represented both early and late apoptotic cells.

### Cell proliferation assay by CCK‐8

Cell proliferation was examined by a Cell Counting Kit‐8 (CCK‐8, APExBIO Company, Houston, TX, USA). After the cells were collected with different collecting buffers, 2000 cells were seeded in each well of a 96‐well plate. We measured the OD450 value by an M5 microplate reader (Molecular Devices, LLC., San Jose, CA, USA) at 24 and 48 h.

### Cell proliferation assay by single‐cell sorting of 96‐well plates

Before sorting, 96‐well plates (Corning) were prepared by adding 200 μL of culture medium to each well. Jurkat cells were suspended in PBS. In this test, one cell was sorted into one well of the plate using three drop delay values. The three drop delay values included the optimal drop delay obtained by manual adjustment, and the other two drop delay values were obtained by adding and subtracting 0.05 from the optimal value. We tested all wells in the plate using each drop delay value and repeated one plate. After sorting, we placed the plate in a 37 °C incubator with 5% CO_2_ for 1 week. We counted the numbers of clones using microscopy.

### Gene sequencing

For some experiments, it is necessary to purify cells for gene sequencing by FACS; therefore, we investigated whether adding FBS to the collecting buffer affects the gene expression characteristics of sorted cells. We established two groups: the PBS group and the 5% FBS in PBS group. Jurkat cells were sorted into the PBS and 5% FBS in PBS buffers. Then, 1 × 10^6^ cells were sorted in 5 min and collected to extract total RNA. A library was built after enrichment purification using magnetic beads with oligo (dT), and a quality library was sequenced via the Illumina platform with a pe150 sequencing strategy. The difference expression multiple was 2. The *P*‐value was 0.05, and *P*adj was 0.05. To detect the difference in gene function caused by sorting, GO and KEGG were used to analyze the sequencing results.

### Statistical analysis

Data are presented as the mean ± SEM and are representative of three independent experiments. Statistical analysis was performed with graphpad prism 6 software (GraphPad Software, San Diego, CA, USA). One‐way ANOVA with the Newman–Keuls *post hoc* test or two‐way repeated measures ANOVA with the Bonferroni *post hoc* test was used for comparison of multiple groups. Significance was set at *P* < 0.05.

## Results

### Effects of drop delay on yield and purity

We attempted to collect cells with the highest yield using a jet‐in‐air sorter. To avoid cell loss due to failure or nonoptimal calibration of the instrument, we decided to modify the drop delay value using a manual method on an automatic basis to find the accurate drop delay value. The result was that the sort yield was averagely 88.4% according to the automatic drop delay value, and the yield reached averagely 94.0% when the drop delay value was modified by the manual method under the same conditions (Fig. 1A). We further analyzed the correlation between drop delay and yield. We confirmed that the drop delay clearly affected the sort yield. For example, when the drop delay was 36.64, the yield was 97.56 ± 1.26%. When the drop delay was slightly increased from 36.64 to 36.84, the yield decreased sharply to 82.67 ± 5.81% (Fig. 1B). In addition, if the time duration of the sorting process was longer, for example, reaching 4 h, we could set the drop delay in an optimal setting model to improve the yield rate by 3–5% (Fig. 1C). Estimated drop delay displayed the paremeters in Fig. 1G.

Then, according to the R‐max method provided in a previous report [7], the two‐way separation experiment was simulated by CST beads to determine the effect of drop delay on the yield and purity of sorted beads. Under the optimal conditions, the R‐max value was 90.8 ± 8.2%. Similarly, the R‐max value significantly decreased when the drop delay was offset. However, the purity remained constant and at a high level of almost 100% regardless of the drop delay value (Fig. 1D,E).

For the living cell experiment, we confirmed the relationship between drop delay and yield by sorting cells into 1536‐well plates (Corning). The effect of drop delay on the purity of Jurkat cells was examined by bulk sorting. The yield rate was 46% when the manual drop delay value was 32.3. The yield rate decreased to 20% when the drop delay value was shifted to 32.6. The change in the drop delay value had no effect on the sorted cell purity, which was almost 100% (Fig. 1F).

In the 96‐well plate test, the average yield was 87.50 ± 3.76% under the manual drop delay conditions, and the yield decreased to approximately 52% when the drop delay values were offset by 0.05 (Fig. S2). Therefore, we can obtain the highest yield by manually adjusting the drop delay.

### Effects of FBS in loading buffer on cell apoptosis

**Fig. 2:**
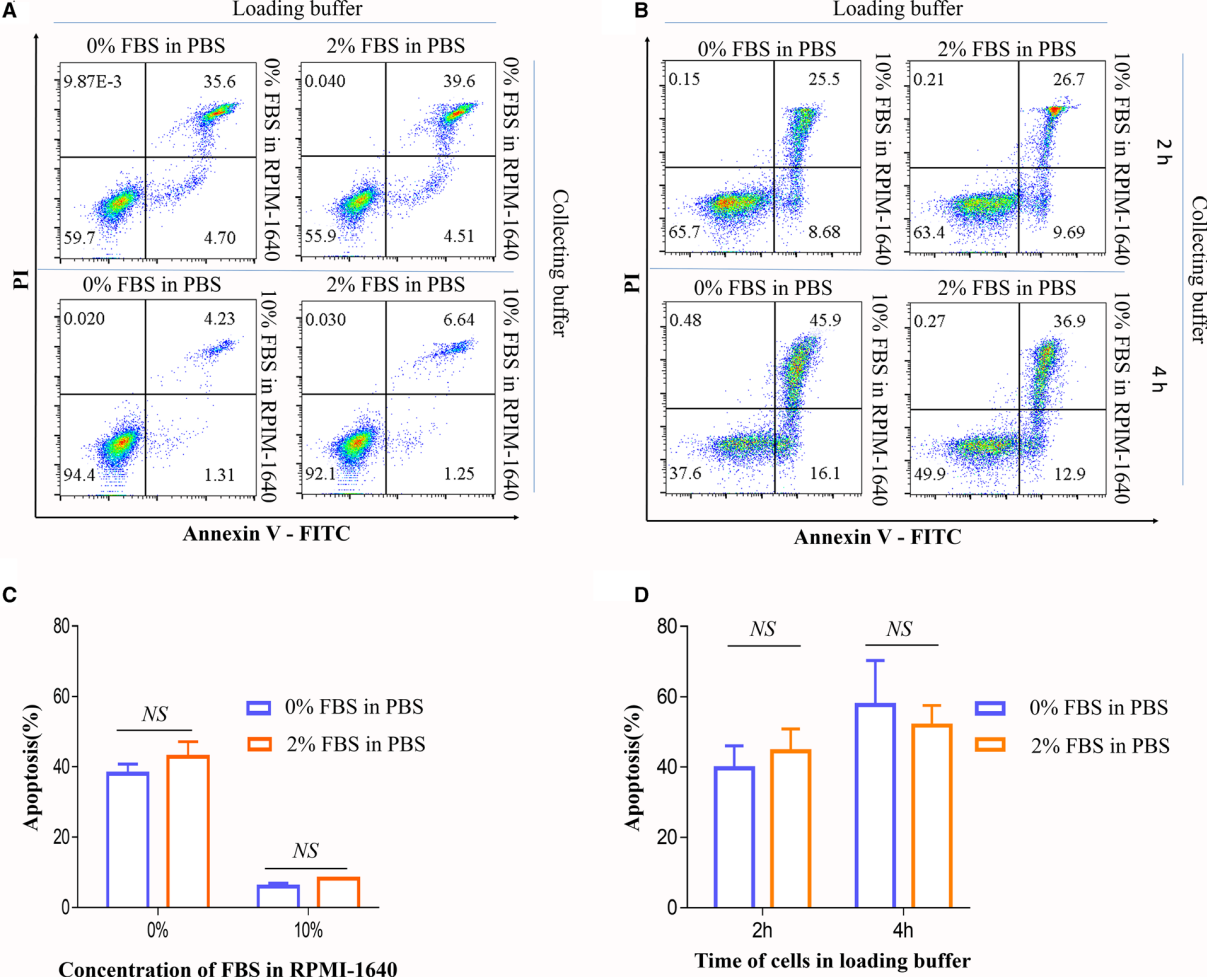
Effects of the addition of FBS in loading buffer on cell apoptosis. (A) Dot plots of apoptotic cells sorted with different loading and collecting buffers. (B) Dot plots of apoptotic cells sorted with different loading and collecting buffers after 2 and 4 h. Numbers in quadrants indicate the percentages of cells in each throughout. Cells were stained with Annexin‐V and propidium iodide and analyzed by flow cytometry. (C, D) The data of the percentage of apoptotic cells of three independent experiments. Data are presented as the mean ± SEM. *n* = 3. Each experiment was repeated at least three times. NS: no significance, by one‐way ANOVA.

First, to explore the effects of FBS in loading buffer on cell viability, we suspended cells with PBS either without FBS or with 2% FBS in loading buffer. Then, the cells were sorted into the collecting buffer, which was RPMI‐1640 medium with or without 10% FBS, on the optimal drop delay. When the collecting buffer was RPMI‐1640 medium only, the apoptosis percentages were 38.05 ± 2.19% in the PBS loading buffer group and 42.85 ± 3.84% in the 2% FBS PBS loading buffer group. When the collection buffer was RPMI‐1640 medium with 10% FBS, the apoptosis percentages were 5.94 ± 0.78% in the PBS loading buffer group and 8.14 ± 0.18% in the 2% FBS PBS loading buffer group (Fig. 2A). These data demonstrated that there was no difference between the loading buffers with or without FBS when the cells were collected with the same collecting buffer (Fig. 2C).

Second, we explored whether the incubation time of cells in loading buffer with or without 2% FBS will affect the cell viability. CD45^+^ cells were incubated in 2% FBS PBS or PBS at 4 °C for 2 and 4 h, respectively. After incubation, cells were checked for the apoptosis rate after sorting. In 2% FBS PBS loading buffer, the apoptosis percentage was 44.49 ± 6.36% at 2 h and was 52.12 ± 6.26% at 4 h. In PBS loading buffer, the apoptosis percentage was 39.61 ± 6.44% at 2 h and was 53.25 ± 6.45% at 4 h. These data demonstrated that the apoptosis rate increased with the time of sorting, but there was no difference in apoptosis rate between 2% FBS PBS and PBS loading buffer at 2 and 4 h (Fig. 2B,D) However, it was clear that the cell apoptosis percentage was significantly decreased by adding FBS to the collection buffer.

### Effects on cell count caused by FBS or BSA in the collecting buffer

On the optimal drop delay and the same other sorted conditions, we suspended cells with PBS and sorted the same cells into a series of collecting buffers. To evaluate the cell viability after sorting, we tested the cell counts, cell apoptosis, and cell proliferation under each condition.

**Fig. 3:**
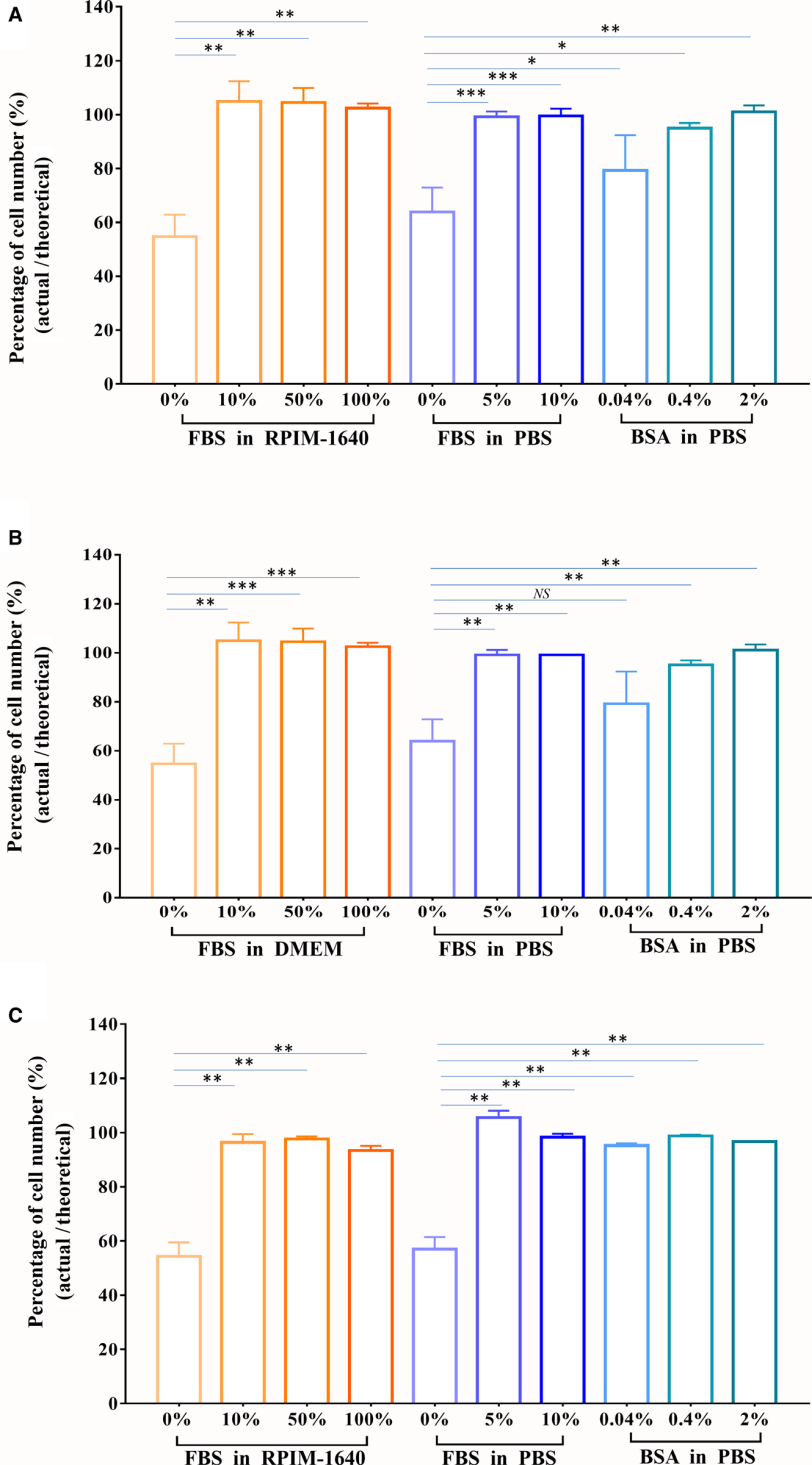
Effects of the additions of FBS/BSA in collecting buffer on cell counts. (A) Jurkat cells. (B) 293T cells. (C) CD45^+^ cells from mouse spleen. Cell counts for sorted cells using different collecting buffers. We collected 500 000 cells using each collecting buffer. A 50 μL cell suspension was tested by flow cytometry. The actual cell amounts were counted. Based on the total cell amounts and weight difference before and after sorting to estimate the theoretical cell amounts in 50 μL collecting buffer, the recovery rate was obtained by the actual cell amounts/the theoretical cell amounts. Data are presented as the mean ± SEM. *n* = 3. Each experiment was repeated at least three times. *P < 0.05, **P < 0.01, ***P < 0.001, NS: no significance, by one‐way ANOVA.

The sorted cells were collected with different collecting buffers followed by direct detection of the cell amounts through flow cytometry. The results showed that the actual values of six kinds of collecting buffers were close to the theoretical value, and the yield rate was close to 100%, including for 5% FBS in PBS, 10% FBS, and 50% FBS in medium, 100% FBS, 0.4% BSA, and 2% BSA in PBS. For Jurkat cells and 293T cells (Fig. 3A,B), the yield rate was at the range of 92.5–104.8%. The yield rate of 0.04% BSA in the PBS group was 83.7 ± 11%, and the worst group was PBS and medium, exhibiting values of 57.85 ± 14.2% and 60.3 ± 10.2%, respectively. For primary cells, CD45^+^ cells from mouse spleen (Fig. 3C), the yield rate was about 93.5–105.5%, and the worst group was also PBS and medium, exhibiting values of 56.99 ± 6.25% and 54.29 ± 7.31%. In terms of the deviation between the theoretical cell amounts and the actual cell amounts, the amounts decreased more than 40% when a collecting buffer without FBS or BSA was employed, while the amounts were almost the same when collecting buffers with FBS at the tested concentration or with 2% BSA were employed. The performance of the cell collection buffer was tested by sorting Jurkat cells, 293T cells, and CD45+ cells, and we obtained similar results. The specific data of Jurkat cells are shown in Table 1.

**Table 1:** Effects of the additions of FBS/BSA in collecting buffer on cell counts and apoptosis for Jurkat cell.

Collecting buffer	FBS in PBS		BSA in PBS			FBS in RPIM‐1640			
	0%	5%	0.04%	0.40%	2%	0%	10%	50%	100%
Actual amounts (50 µL)	6135	9272	7903	8971	9506	5715	9733	9864	10 299
Theoretical amounts (50 µL)	9085	9330	9860	9697	9609	9526	9519	9556	9819
Apoptosis (%)	34.44 ± 6.28	6.95 ± 0.41	9.61 ± 0.52	10 ± 0.61	8.73 ± 0.63	38.05 ± 2.19	5.94 ± 0.78	6.42 ± 1.29	6.14 ± 0.13

The significance of underline indicates the effect of additions.

### Effects of FBS or BSA in collecting buffer on cell apoptosis

**Fig. 4:**
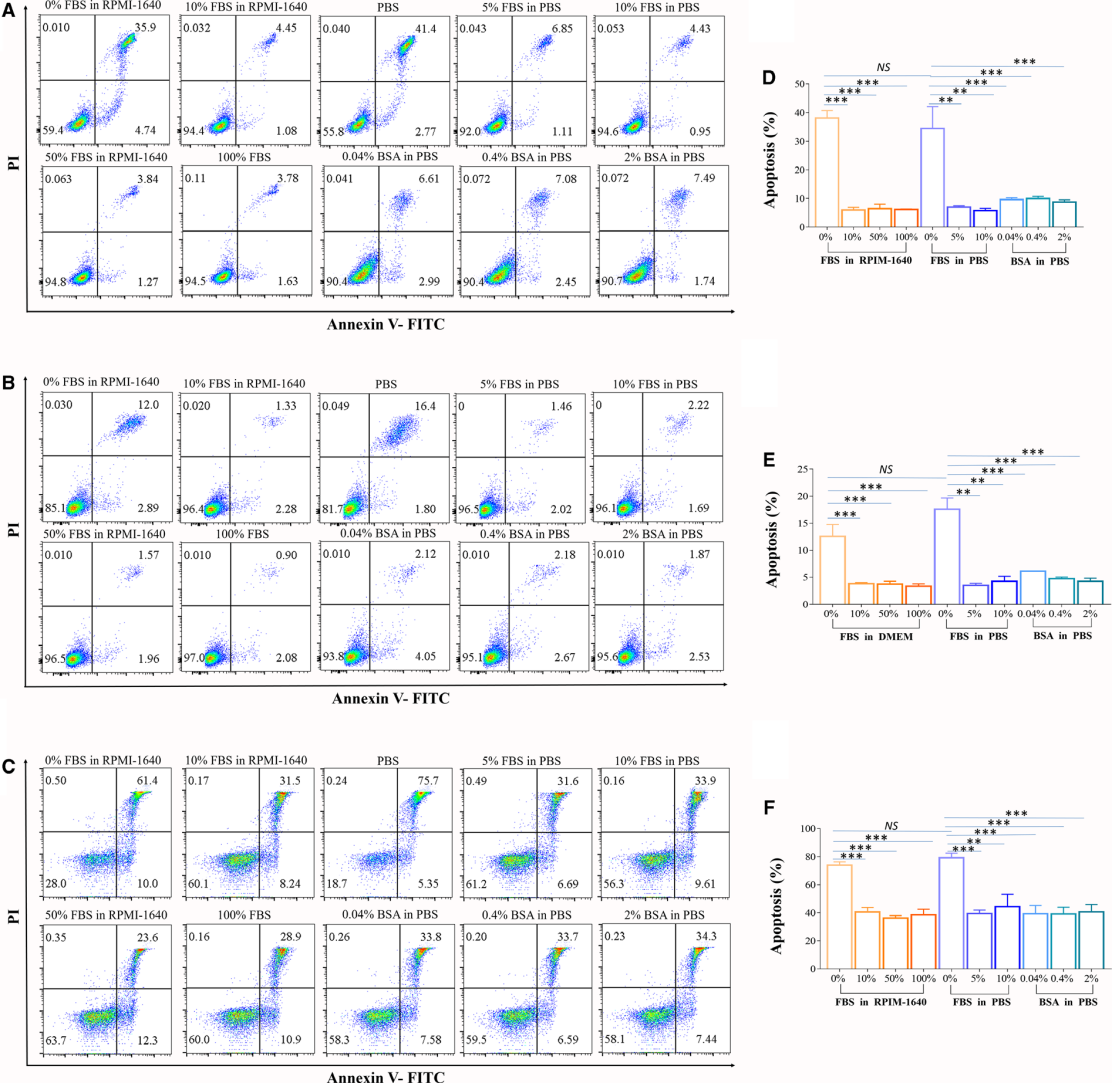
Effects of the addition of FBS/BSA in collecting buffer on cell apoptosis. (A–C) Dot plots of apoptotic cells sorted with different collecting buffers. Numbers in quadrants indicate the percentages of cells in each throughout. Cells were stained with Annexin‐V and propidium iodide and analyzed by flow cytometry. A is Jurkat cells, B is 293T cells, and C is CD45^+^ cells from mouse spleen. (D–F) The concentration of FBS or BSA in the collection buffer affects the apoptotic rate. The data of the percentage of apoptotic cells shown in D–F are the means of three independent experiments. D is Jurkat cells, E is 293T cells, and F is CD45^+^ cells from mouse spleen. Data are presented as the mean ± SEM. *n* = 3. Each experiment was repeated at least three times. **P < 0.01, ***P < 0.001, NS: no significance, by one‐way ANOVA.

We next assessed the apoptosis rate after sorting with different collecting buffers. Figure 4 shows the apoptosis rates of Jurkat cells (Fig. 4A,D) and 293T cells (Fig. 4B,E). For Jurkat cells, the apoptosis rates of cells collected in PBS and RPMI‐1640 medium were 34.4 ± 6.28% and 38.1 ± 2.19%, respectively. The corresponding apoptosis rates of PBS with 5% FBS and 10% FBS were 6.95 ± 0.4% and 5.73 ± 0.6%, respectively. The apoptosis rates of 10% FBS, 50% FBS, and 100% FBS in the medium groups were 5.94 ± 0.78%, 6.42 ± 1.3%, and 6.14 ± 0.13%, respectively. The apoptosis rates of 0.04%, 0.4%, and 2% BSA in the PBS groups were 9.61 ± 0.5%, 10 ± 0.6%, and 8.7 ± 0.6%, respectively. The data of Jurkat cells showed that the collecting buffer with FBS or BSA can maintain high cell viability with apoptosis less than 10%, while the collecting buffer without FBS or BSA increased apoptosis by almost 40%, regardless of medium or PBS. The apoptosis performance of the collecting buffer was also tested by sorting 293T cells and hESC‐MSCs (Fig. S3). We obtained similar results. It should be noted that the apoptosis rate of 293T cells was lower than that of the other tested cells. The apoptosis rate of PBS was 17.59 ± 1.7%, and the apoptosis rate of medium was 12.6 ± 1.8%. The apoptosis rate in the 0.04% BSA group was 6.13 ± 0.06%. However, the apoptosis rate in the other groups was less than 5%. These results indicated that FBS or BSA in collecting buffers had protective effects on the apoptosis of sorted cells. We got the same results on the BD ARIR II (data not shown).

For CD 45^+^ primary cells, the apoptosis rates in PBS and RPMI‐1640 medium were 79.25 ± 3.33% and 73.88 ± 2.37%, respectively. While the apoptosis rates in 5% FBS and 10% FBS PBS groups were 39.43 ± 2.50% and 44.33 ± 8.78%, respectively. Moreover, the apoptosis rates in 10% FBS, 50% FBS, and 100% FBS RPMI‐1640 medium groups were 40.58 ± 3.19%, 36.2 ± 1.77%, and 38.52 ± 4.04%, respectively. The apoptosis rates in 0.04%, 0.4%, and 2% BSA PBS groups were 39.27 ± 5.99%, 39.03 ± 4.97%, and 40.6 ± 5.32%, respectively (Fig. 4C,F). The data showed that the collecting buffer with FBS or BSA can maintain primary cell viability with lower apoptosis rate (average 40%) also. The apoptosis rate of primary cells after sorting was significantly higher than that of cell lines.

### Effects of FBS in collecting buffer on cell proliferation

**Fig. 5:**
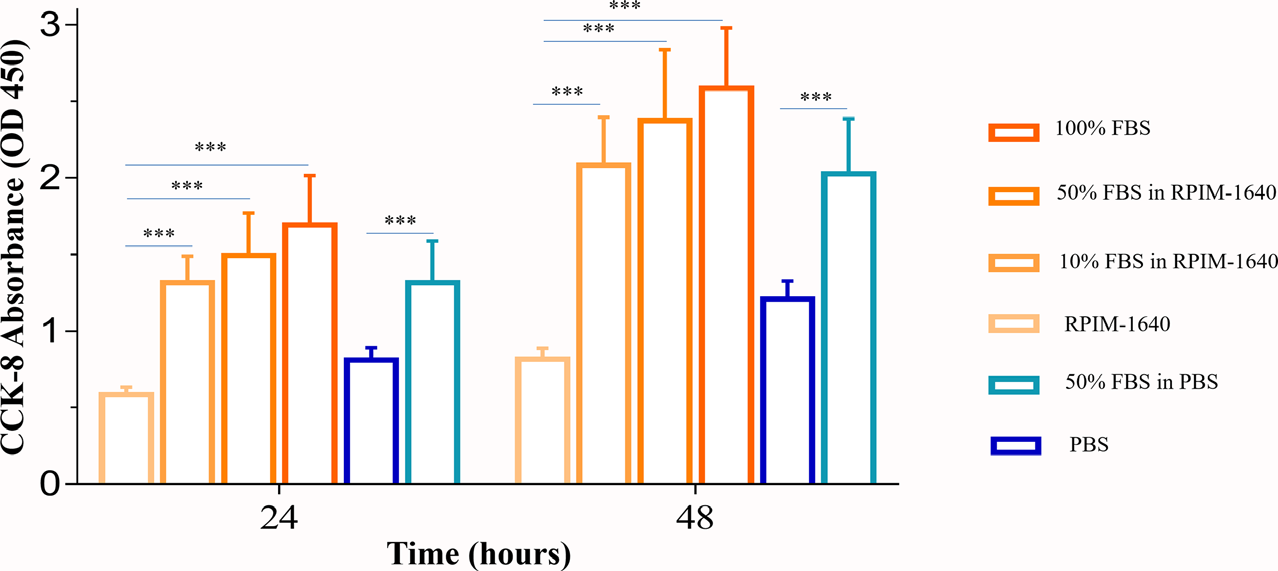
Effects of the addition of FBS/BSA in collecting buffer on cell proliferation. Each collecting solution contained 2000 cells in each well. Cells were incubated for 24 and 48 h. Cell proliferation was assessed by CCK‐8 assay. Data are presented as the mean ± SEM. *n* = 3. Each experiment was repeated at least three times. ***P < 0.001, NS: no significance, as determined by two‐way ANOVA.

We further cultured the sorted Jurkat cells for 48 h, and the growth curve showed that the cells collected by RPMI‐1640 with FBS had strong proliferation regardless of the FBS concentration that was used (Fig. 5)

### Collecting buffer with the presence of 5% FBS can stabilize the genome expression of sorted cells

**Fig. 6:**
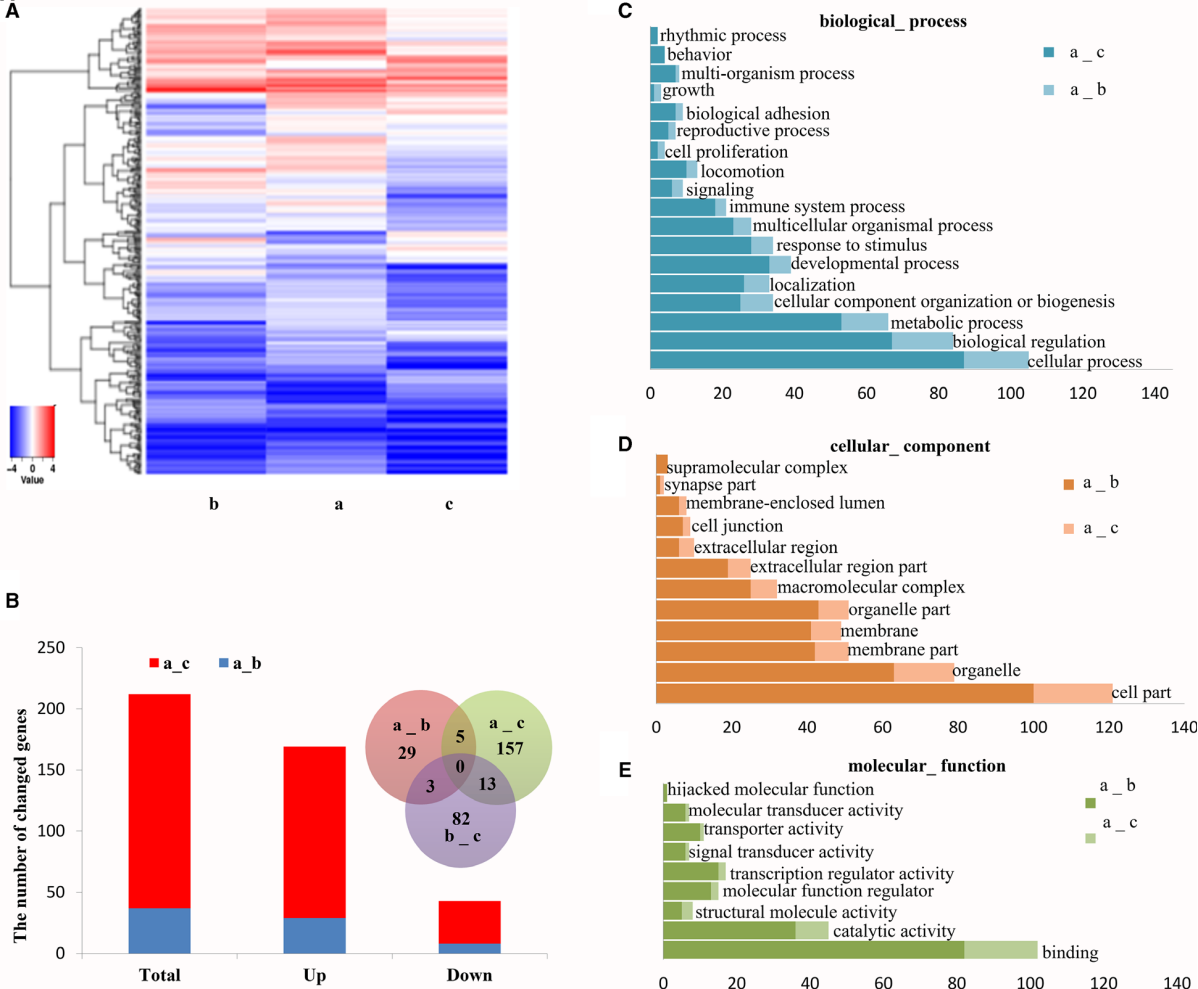
Effects of FBS in collecting buffer on mRNA expression in Jurkat cells. (A) Hierarchical clustering analysis of unsorted cells and sorted cells were harvested using collecting buffer with or without FBS. The expression levels of genes (fold change > 2.0, *P* < 0.05) are presented in different colors, indicating expression levels above and below the median expression level across all samples. (B) The number of genes that were up‐ or downregulated of cells collected using PBS or 5% FBS in PBS compared to unsorted cells. Venn diagram showed the overlap of genes with abnormal expression using different collecting buffers. (C–E) GO annotations of affected genes from different collecting buffers. The bar plot presents the numbers of affected genes of significantly enriched GO terms in biological processes, cellular components, and molecular functions. a: unsorted cells; b: sorted cells collected using 5% FBS in PBS; c: sorted cells collected using PBS.

To determine the effects of FBS in the collecting buffer on gene expression, we sorted Jurkat cells into collecting buffers consisting of PBS either without FBS or with 5% FBS. We harvested RNA from the cells before and after sorting. The results showed that the gene expression of sorted cells collected with 5% FBS in the collecting buffer was closer to that of unsorted cells (Fig. 6A, Database ID: SUB8446820). Compared to unsorted cells, cells collected using PBS alone showed 175 up‐regulated genes and 35 downregulated genes. In the presence of 5% FBS in PBS, the number of affected genes was sharply decreased. There were only 29 upregulated genes and 8 downregulated genes. The Venn diagram shows the correlation of affected genes among each group (Fig. 6B). The GO annotations of affected genes showed that these genes were involved in a wide range of cell functions in biological processes, cellular components, and molecular functions (Fig. 6C–E). The top five up‐ and downregulated genes after sorting using different collecting buffers are listed in Tables S1 and S2. The data demonstrated that the inclusion of 5% FBS in the collecting buffer significantly protected the inherent gene expression of Jurkat cells during sorting.

## Discussion

An accurate drop delay assessment is critical to achieve successful cell sorting with high purity and yield [12]. Depending on the desired output, the target population may be sorted using three different modes: enrich, purify, and single cell. When recovery is the most important aspect of the sort, the enrich mode is used. The purify mode is used when purity is as important as recovery. When the most important aspect of the sort is that one drop contains one particle or one cell, the single mode is used. We used ‘purify’ and ‘single cell’ sort mode in our study. Our results were consistent with the conclusion of previous study, demonstrating that when sorting in purify or single‐cell modes, yield is still the primary criterion to assess the performance of a cell sorter. Since factors affecting purify will compromise recovery, issues compromising recovery may not necessarily affect purity [7].

During the process of a jet‐in‐air cell sorting experiment, the droplets which including the target particle can be accurately sorted into collection tubes depending on the drop delay, while other droplets pass into the waste. The drop delay value, which is invariably the most critical sorting parameter, is defined as the distance in time between the laser interrogation point and droplet breakoff point (expert.Cheekyscientist.com) (Fig. S4). Thousands of individual droplets (as many as 90 000 or more) will be generated by vibrating the stream, and each droplet is supposed to encompass a cell [13]. In addition, the sample‐loading velocity (eps) is about 1/4–1/5 of the vibration frequency. Therefore, the empty droplet before and after the target droplet is dominant and the delay deviates in a certain range will subsequently result in the unexpected mixture of empty and target particle droplets. Thus, the empty droplet is sorted, which leads to the decrease of the yield but with little influence on the purity.

Our results demonstrate that the yield of sorting was improved by manually adjusting the drop delay. This approach was also used in the analysis of beads and cells, and the results were better than those of previous reports [14]. The results demonstrated that a precise drop delay can obtain the highest sorting yield and purity. Deviation of drop delay in a narrow region can maintain perfect purity but sharply decrease yield. In other words, yield is considerably more sensitive to the deviation of drop delay than purity is. It was sure that a drop delay should perform exactly the same regardless of which method was used to determine it. In our study, we only want to indicate that the accuracy of drop delay value can be verified manually, and in the case of not accurate, delay can be manually adjusted to obtain a better value. Figure 1a shows that sometimes you cannot acquire accurate drop delay values under auto mode (red), but you can acquire accurate drop delay values under manually mode (blue). The nonaccurate drop delay by auto mode mainly attributes to the inappropriate setting for the values of defanning and charge phase during drop delay regulation, especially for beginners. Therefore, manual adjustment is not necessary for every experiment, especially for experienced instrument operators. The results suggest that the droplet delay is better to be readjusted before sorting single cells, rare cells, or fragile cells, manually.

However, in an experiment on long‐term sorting, the drop delay may be shifted slightly due to the status of the sorter changing, even if the sorter can still maintain sorting. This finding may be attributable to the decrease in sorting yield of approximately 3–5% (Fig. 1C). Petersen *et al*. [15] found that small variations in the pressure and drive amplitude can lead to correspondingly large changes in the breakoff time. They had shown that the pressure in the tank varies by as much as 3% depending on whether the sheath reservoir is full or empty. In our study, after 3–4 h of sorting, about half the volume of sheath in the tank could be consumed, which is enough to cause changes in pressure. Moreover, they also indicated that breakoff time changes even if the change which in the distance to the breakoff point was corrected by piezoelectric oscillation amplitude. The results of our study showed the changes from the above two points can affect the yield of sorting in a small range (3–5%), but not enough to affect the purity of sorting. Therefore, after a long time of sorting (*t* ≥ 3 h), if there is a higher requirement for sorting yield such as single cell and rare cell sorting, it is recommended to recalculate the drop delay value. In this study, we suggest that if a single cell needs to be sorted after continuous sorting for 3–4 h, it is better to recalculate the drop delay to obtain a better yield. For most FACS sorting, especially for the sorting of single cells, rare populations, or precious samples, the primary goal is to obtain as many cells of interest as possible based on good purity. Therefore, guaranteeing a precise drop delay during sorting is indispensable for obtaining a high sorting yield. In addition, experienced instrument operators can obtain similar to manual mode drop delay through automatic mode, similar to the result in the study, but for beginners, because of the uncertainty increase, manual adjustment is more essential.

The R‐max method holds that evaluating the actual recovery of a given sort typically relies on direct measurements of the absolute number of target particles in the sorted and original fractions. To validate whether the manual method makes the equipment with the highest yield, R‐max values on the manually modified drop delay were compared with those that were reported in the article. The data showed that the R‐max value with the manually modified drop delay was higher. By manually adjusting the drop delay described in this paper, the instrument can achieve the highest yield.

The decrease in cell viability during sorting may primarily due to the pressure and strong electric field associated with the sorting process [16, 17]. Our results showed that 2% FBS in the loading buffer had almost no effect on yield and purity in 4 h. We hypothesized that this lack of effect might be observed because the cells are not damaged by the sorting process; in fact, the cells were healthy. Therefore, utilizing loading buffer with or without FBS might have little influence on cell conditions. However, importantly, we did observe that including FBS in the collecting buffer does have a strong influence on the viability of cells, rather than that in the loading buffer. The addition of 10% FBS or 2% BSA in the collecting buffer considerably increased the cell amounts, decreased apoptosis, and promoted the proliferation of sorted cells (Table 1).

In recent years, many important cellular characteristics have been assessed specifically with the help of single‐cell approaches. High‐throughput single‐cell transcriptomics has provided unprecedented insights into the cellular diversity of tissue. For this method, maintaining gene expression is critical. Graham M. Richardson used a microarray to show that the selection of the sorter instruments was considerably less important than other factors related to how cells are isolated and handled with regard to short‐term gene expression [18]. However, our data showed that including FBS in the collection buffer would have an effect on gene expression. Paired analysis of gene expression showed significant differences between the two groups. These results suggest that adding 5% FBS to the collecting buffer is as important as maintaining a temperature of 4 °C to minimize the gene expression changes through the duration of the sorting process [18, 19].

‘Garbage in, Garbage out’ is suitable for all sorting experiments, so the state of the cells before sorting plays a decisive role in addition to the factors mentioned in the article that affect the viability. For 96‐well plate single‐cell sorting, the position of the plate is also a key factor to be considered.

**Fig. 7:**
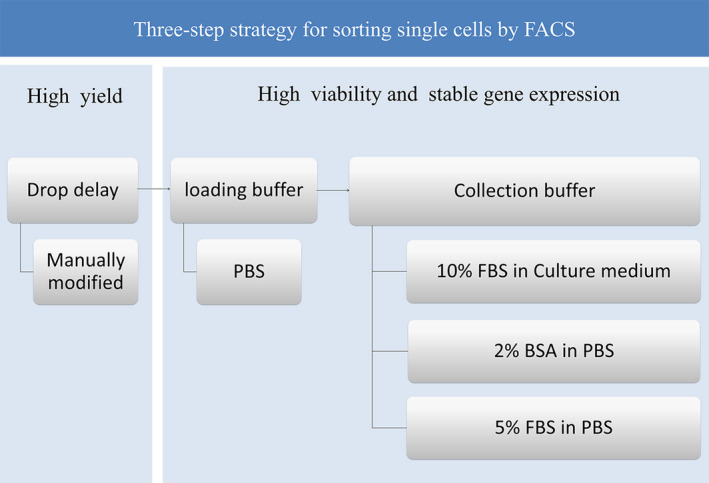
Three‐step strategy for sorting single cells by FACS. First step: set the drop delay by manually modifying the model to obtain a high yield. Second step: PBS could be used as loading buffer, which has no significant effect on cell viability in 4 h. Third step: 10% FBS in culture medium, 2% BSA in PBS, or 5% FBS in PBS could be selected as the collecting buffer depending on the different requirements of the experiment. Specifically, 5% FBS in PBS is optimal for single‐cell RNA sequencing because it affects gene expression less than PBS alone.

Our results strongly suggest that the concentration of FBS or BSA in the collection buffer was crucial for downstream experiments, including cell culture and genomic expression experiments. We recommend adding 5% FBS, 10% FBS, or 2% BSA in the collecting buffer, either in PBS or in culture medium, depending on the experimental design (Fig. 7).

In summary, our study demonstrates that an effective strategy can be employed to perform sorting with high yield, viability, and gene expression stability. Furthermore, researchers may adjust the collecting buffer for sorting based on their downstream experiments and the results of these modifications to cell sorting parameters, which have exhibited notable improvements with the minor adjustments that we made, and need not worry about any problems related to collecting or loading buffer. Furthermore, adding extra expensive FBS in collecting or loading buffer to boost yield is no longer necessary. In all, this will make the downstream experiments smoother.

## Conflict of interest

The authors declare no conflict of interest.

## Author contributions

XS, HH, and LW conceived and designed the project, XS, LX, YX, YL, CG, and YH acquired the data, XS and JW analyzed and interpreted the data, XS and LW wrote the paper.

## Supporting information

**Fig. S1**. The drop delay Diagram and Estimated drop delay. The drop delay Diagram showed how the puddles (the circles) are deposited on the slide, and the criterion of automatic and manual drop delay. The 5# puddle contains the most beads, the difference between the number of beads in the 4# and 6# puddles adjacent to 5# puddle is less than 3% for the auto mode, but the criterion of manual mode is that there is no bead in both 4# and 6# puddles. Estimated drop delay displayed the parameters of the two modes.

**Fig. S2**. The yield of cells was assessed under manually adjusted conditions. Jurkat cells was sorted into one well of the plate using three drop delay values. All wells were tested in the plate using each drop delay value and repeated one plate. After sorting, the plate was placed in a 37°C incubator with 5% CO2 for one week. The numbers of clones were counted using microscopy.

**Fig. S3**. Effects of the addition of FBS in collecting buffer on cell apoptosis of human ESC‐ MSC. The concentration of FBS in the collection buffer affects the apoptotic rate. The data of the percentage of apoptotic cells shown are the means of three independent experiments. Data are presented as the mean ± SEM. n=3. ***p < 0.001, by one‐way ANOVA.

**Fig. S4**. The process of the jet‐in‐air sorting. During the process of a jet‐in‐air cell sorting experiment, the droplets which including the target particle can be accurately changed depends on the drop delay, and fall into collection tubes, while uncharged droplets pass into the waste. The drop delay value, which is invariably the most critical sorting parameter, is defined as the distance in time between the laser interrogation point and droplet breakoff point.

**Table S1**. Top 5 up‐ and down‐regulated genes between unsorted cells and sorted cells collected in 5% FBS in PBS buffer.
**Table S2**. Top 5 up‐ and down‐regulated genes between unsorted cells and sorted cells collected in PBS buffer.

## Data Availability

The data that support the findings of this study are presented in the figures, tables, and supplementary information of this article.
