# Supplementary figures and images for: Improved strategy for jet‐in‐air cell sorting with high purity, yield, viability, and genome stability
Source: FEBS Open Bio. 2021 Jul 30;11(9):2453–67. doi: 10.1002/2211-5463.13248 (PMC8409286; doi:10.1002/2211-5463.13248)

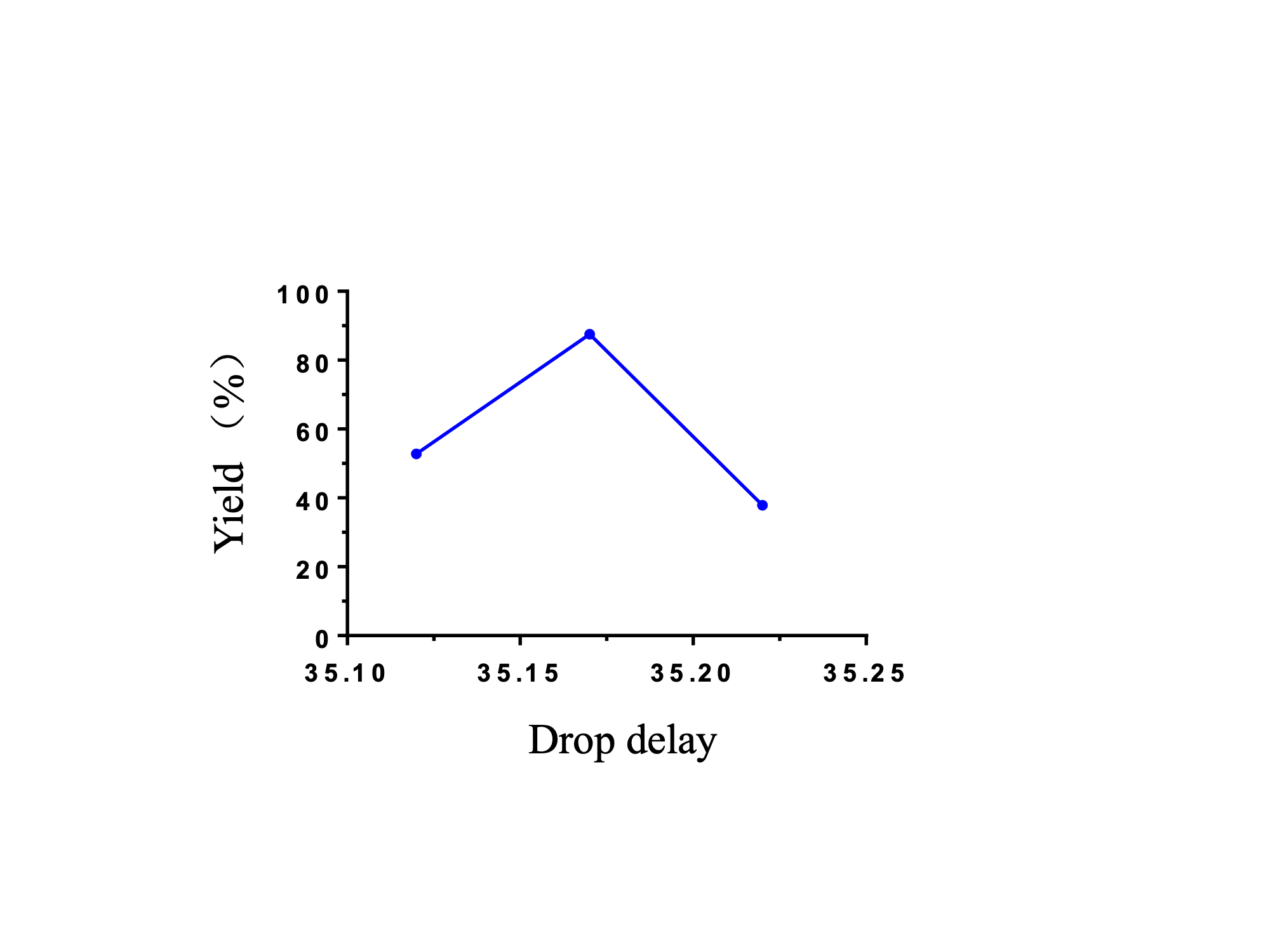

Supplement: Supplementary file 1 — Fig. S1. The drop delay Diagram and Estimated drop delay. The drop delay Diagram showed how the puddles (the circles) are deposited on the slide, and the criterion of automatic and manual drop delay. The 5# puddle contains the most beads, the difference between the number of beads in the 4# and 6# puddles adjacent to 5# puddle is less than 3% for the auto mode, but the criterion of manual mode is that there is no bead in both 4# and 6# puddles. Estimated drop delay displayed the parameters of the two modes. [file FEB4-11-2453-s005.tiff]

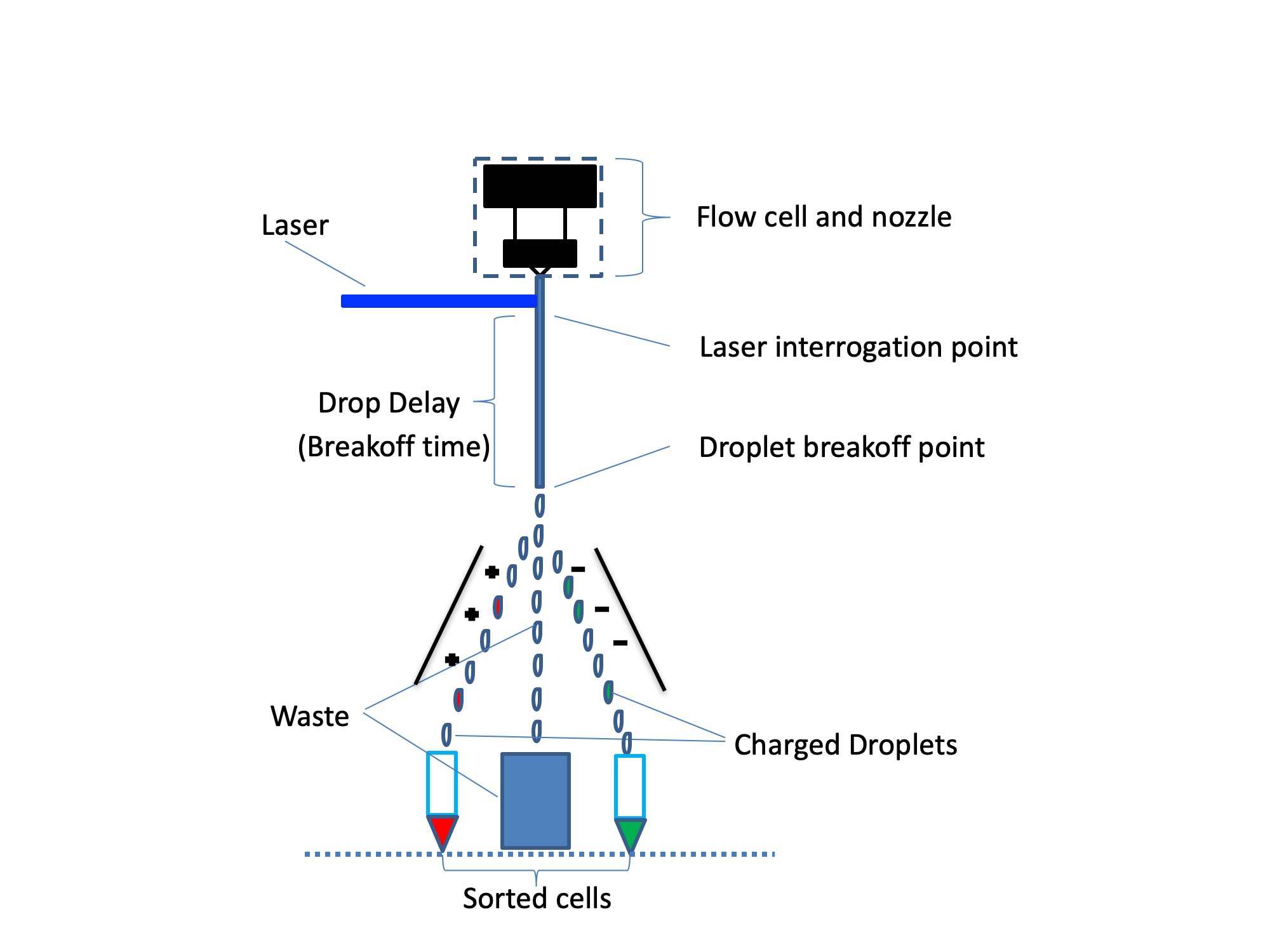

Supplement: Supplementary file 2 — Fig. S2. The yield of cells was assessed under manually adjusted conditions. Jurkat cells was sorted into one well of the plate using three drop delay values. All wells were tested in the plate using each drop delay value and repeated one plate. After sorting, the plate was placed in a 37°C incubator with 5% CO2 for one week. The numbers of clones were counted using microscopy. [file FEB4-11-2453-s002.tiff]

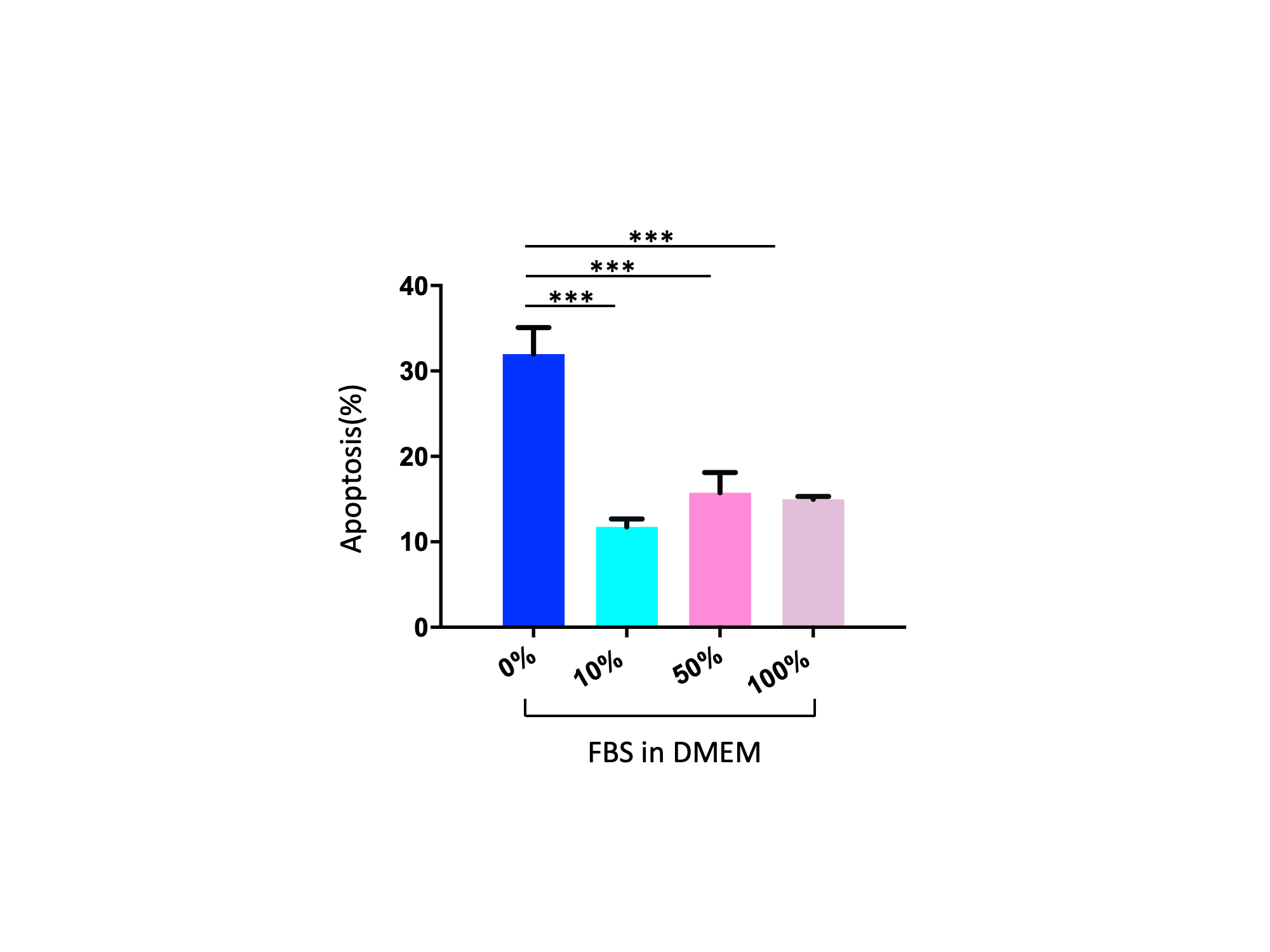

Supplement: Supplementary file 3 — Fig. S3. Effects of the addition of FBS in collecting buffer on cell apoptosis of human ESC‐ MSC. The concentration of FBS in the collection buffer affects the apoptotic rate. The data of the percentage of apoptotic cells shown are the means of three independent experiments. Data are presented as the mean ± SEM. n=3. ***p < 0.001, by one‐way ANOVA. [file FEB4-11-2453-s003.tiff]

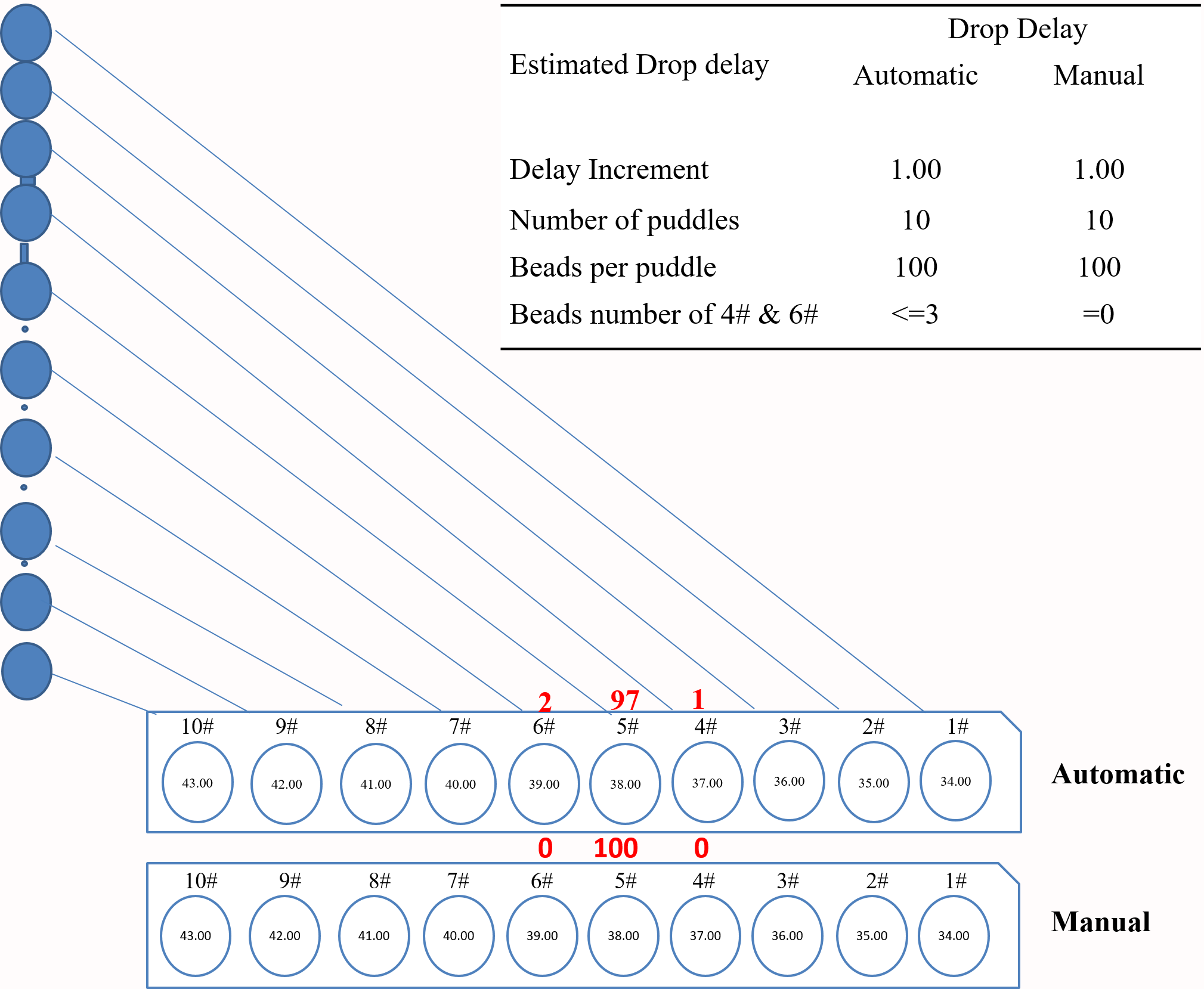

Supplement: Supplementary file 4 — Fig. S4. The process of the jet‐in‐air sorting. During the process of a jet‐in‐air cell sorting experiment, the droplets which including the target particle can be accurately changed depends on the drop delay, and fall into collection tubes, while uncharged droplets pass into the waste. The drop delay value, which is invariably the most critical sorting parameter, is defined as the distance in time between the laser interrogation point and droplet breakoff point. [file FEB4-11-2453-s004.tiff]
