# Supplementary material for: Improved strategy for jet‐in‐air cell sorting with high purity, yield, viability, and genome stability
Source: FEBS Open Bio. 2021 Jul 30;11(9):2453–67. doi: 10.1002/2211-5463.13248 (PMC8409286; doi:10.1002/2211-5463.13248)
Supplement: Supplementary file 5 — Table S1. Top 5 up‐ and down‐regulated genes between unsorted cells and sorted cells collected in 5% FBS in PBS buffer. Table S2. Top 5 up‐ and down‐regulated genes between unsorted cells and sorted cells collected in PBS buffer. [file FEB4-11-2453-s001.docx]

supplementary table S1：Top 5 up- and down-regulated genes between unsorted cells and sorted cells collected in 5% FBS in PBS buffer.

| Gene | FoldChange | Up/Down | GeneName | Position | strand | p-value |
| --- | --- | --- | --- | --- | --- | --- |
| ENSG00000279970 | 35.38 | up | AC023024.2 | chr15 | - | 9.49E-06 |
| ENSG00000270813 | 33.52 | up | NANOGNBP3 | chrX | - | 1.69E-05 |
| ENSG00000230074 | 24.21 | up | AL162231.2 | chr9 | + | 0.000319502 |
| ENSG00000258646 | 22.34 | up | AL049780.1 | chr14 | - | 0.000583781 |
| ENSG00000146521 | 20.48 | up | LINC01558 | chr6 | - | 0.001072487 |
| ENSG00000259943 | 0.49 | down | AL050341.2 | chr1 | - | 2.90E-06 |
| ENSG00000273238 | 0.49 | down | TMEM271 | chr4 | - | 0.003237666 |
| ENSG00000268883 | 0.48 | down | PNMA6B | chrX | - | 0.002885594 |
| ENSG00000204420 | 0.48 | down | MPIG6B | chr6 | + | 0.002301752 |
| ENSG00000212127 | 0.47 | down | TAS2R14 | chr12 | - | 0.000248019 |
|  |  |  |  |  | - |  |

| Gene | FoldChange | Up/Down | GeneName | Position | strand | p-value |
| --- | --- | --- | --- | --- | --- | --- |
| ENSG00000138823 | 26.99 | up | MTTP | chr4 | + | 0.00015 |
| ENSG00000123411 | 15.42 | up | IKZF4 | chr12 | + | 0.000109 |
| ENSG00000242951 | 14.46 | up | AC007182.2 | chr14 | - | 0.000201 |
| ENSG00000253240 | 13.49 | up | IGHV3-36 | chr14 | - | 0.000371 |
| ENSG00000102539 | 13.49 | up | MLNR | chr13 | + | 0.000371 |
| ENSG00000095059 | 0.47 | down | DHPS | chr19 | - | 0.000371 |
| ENSG00000279838 | 0.29 | down | AL356273.3 | chr1 | - | 0.000376 |
| ENSG00000152939 | 0.23 | down | MARVELD2 | chr5 | + | 9.93E-05 |
| ENSG00000278126 | 0.18 | down | AC139768.1 | chr12 | - | 3.84E-06 |
| ENSG00000241923 | 0.13 | down | RPL14P3 | chr4 | - | 0.000685 |

supplementary table S2：Top 5 up- and down-regulated genes between unsorted cells and sorted cells collected in PBS buffer.
